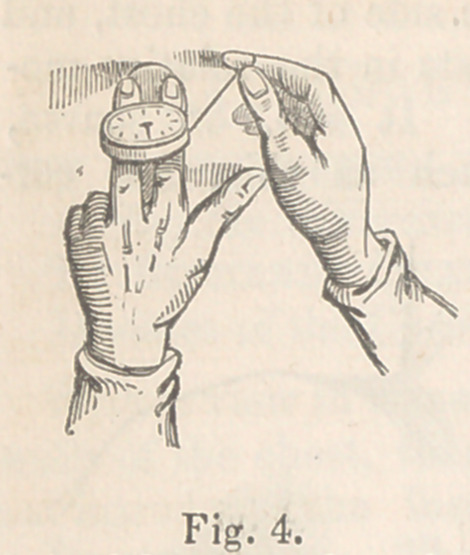# The Stethometer: An Instrument for Facilitating Diagnosis, by Measuring the Difference in the Mobility of Opposite Sides of the Chest

**Published:** 1851-02

**Authors:** Richard Quain

**Affiliations:** Assistant-Physician to the Hospital for Diseases of the Chest at Brompton, etc.


### RECORD OF MEDICAL SCIENCE.

### PATHOLOGY AND PRACTICE OF MEDICINE.

THE STETHOMETER: *An instrument for facilitating diagnosis, by measuring the difference in the mobility of opposite sides of the chest.* By RICHARD QUAIN, M.D., Assistant-Physician to the Hospital for Diseases of the Chest at Brompton, etc.

With a view to a more accurate examination of the movements of the walls of the chest, than can be detected by the unassisted eye, Dr. Quain has introduced the instrument about to be described.

DESCRIPTION. The little instrument (see fig. 1*) consists of a flat case, not unlike a watch case; and on its upper surface is a graduated dial and an index, which stands at a maximum number, that may be considered a "zero." This case contains a very simple movement, by means of which the index can be acted on. A silk cord, which may be of a sufficient length to surround one-half, or the whole circumference of the chest, passes through an aperture in one side of the case. This cord acts on the index. When the cord is drawn out, or extended for the space of one quarter of an inch, it will be observed that the point of the index will once traverse the circumference of the graduated dial. In other words when the index has gone once round, it shows that the cord has been extended one fourth of an inch. It will be further seen, that the dial is graduated, or divided, into fifty equal parts. Each of these parts is, therefore, equal to the fiftieth part of a quarter of an inch; that is, to the 1-200th of an inch.† The index is further capable of going round a second time, on an additional quarter inch of the cord being drawn out. Hence, two revolutions of the index are equal to half an inch of movement—an extent of motion sufficient for all practical purposes. The pedestal and circular foot, shown in the same figure will be subsequently referred to.

Fig. 1.

MODE OF APPLICATION. It is quite evident, that if the instrument be so placed that extension be made on the cord, the amount of the extension will be shown by the movement of the index on the dial. For example (as in fig. 2) if the instrument is laid flat on the spine, and held in its place by the first and second fingers of the left hand, whilst the cord is carried round the chest, and pressed on one of the ribs, or the sternum, by the fingers of the right hand, then, when the individual un-

* The instrument is reduced in this sketch to half size.

† In making and recording observations with this instrument, I am in the habit of expressing a fact thus: right apex, 30; left, 15. It will be understood that the motion is as 15 to 30, without reference to these figures being eighths, fiftieths, or two hundredths.

der examination expands the chest during inspiration, the amount of expansion will be communicated to the cord, and thus indicated on the dial. The cord may then be directed around the opposite side of the chest, and thus will be at once seen any difference which exists in the relative mobility of the two at the point under examination. It will, of course, be absolutely necessary in every examination, such as this, that corresponding parts of the chest be compared. For all useful purposes it may be said, that the movements of the opposite sides of the chest, in health, are identical—the difference which exists over the region of the heart, at the left side, is too immaterial to interfere with practical conclusions. The instrument may be applied to any part of the chest in the mode here described. In figure 3 it is shown as applied on the sternum, and beneath one of the clavicles. The latter position is one of considerable importance, from its connexion with the deposition of tubercle towards the summit of the lung. I find that on applying the instrument here the cord may, in this instance, be directed towards the arm more conveniently than in any other direction, as shown by the black line, and pressed against a point near the insertion of the deltoid muscle. The cord may or be directed downwards, or outwards, and upwards, or inwards, (as shown by the dotted lines,) and retained on any fixed point. Nor, as is evident, need this point be a part of the body. It may be on the bed, or on a chair, etc., always providing that the direction of the cord be such as to receive the impression of the movement of the part of the body under examination, and that its position be symmetrical at both sides.

Fig. 2.

Fig. 3.

The use of the pedestal, which can be fixed in the side of the case with the foot attached, is shown in figure 4. The instrument thus used is intended to ascertain the modifications of the movements over a limited or circumscribed spot. On this spot the foot is placed and held, (as shown in the figure) by the fingers of the left hand, the cord directed towards this point is held between the fingers of the right hand. These fingers being thus made the fixed point, must be kept steady and not allowed to touch the part under examination. The spring in the instrument is sufficiently strong to resist the gentle tension made by the fingers, and the instrument itself being pushed forward or raised by the elevation of the part during inspiration, the movement of the index, as when the case is applied on the flat surface, becomes the measure of this elevation.

Fig. 4.

Such being the mode of using the instrument, it will be necessary to say a few words on the precautions which are required in securing accuracy in the result. 1st. It is absolutely imperative that corresponding portions of the chest be examined, and that the mode of applying the instrument, and the point at which it is applied at each side, be identical. 2nd. Care must be taken that the patient breathes in the same manner whilst opposite sides are being examined. 3rd. It should be seen that the cord, when the observation is commenced, is held sufficiently tense to act on the index.

All this requires attention and some little effort; for, as in all other matters, there is little that is of any value which can be obtained without some labor. Though to some few patients these carefully conducted examinations may be irksome, yet to the vast majority they are far otherwise. In the latter, they beget confidence in the medical attendant, and frequently at once inspire a feeling which renders all future intercourse not less pleasing than it is conducive to successful treatment.

The object of the instrument is not so much to test the relative breathing powers of different individuals, (for testing which, Dr. Hutchinson's spirometer is a more correct means,) but to ascertain any want of symmetry, independently of malformation, and therefore indicative of disease, in the movements of the corresponding parts of the same chest. How much a morbid condition of the lungs may interfere with the free movements of the walls of the chest, is obvious; and in commencing disease—the earlier stages of phthisis for instance—or as a confirmation of the existence of lesion suspected by other means, mensuration will prove a very valuable aid in diagnosis. It need not, of course, be pointed out, that it does not apply when both sides of the chest are equally diseased, a very rare occurrence, however.

The instrument may be also turned to account as a means of measuring increase or diminution in the volume of solid tumours, or other swellings. —*Abridged from London Journal of Medicine, October*, 1850.

## Figures and Tables

**Fig. 1. f1:**
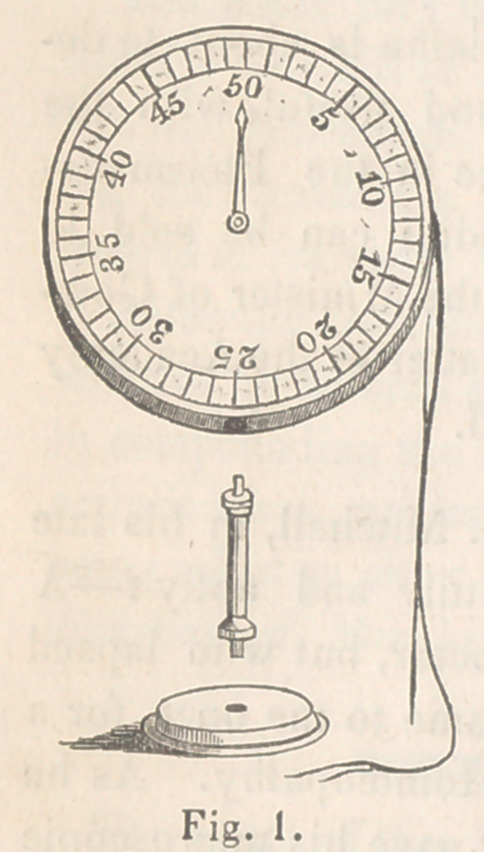


**Fig. 2. f2:**
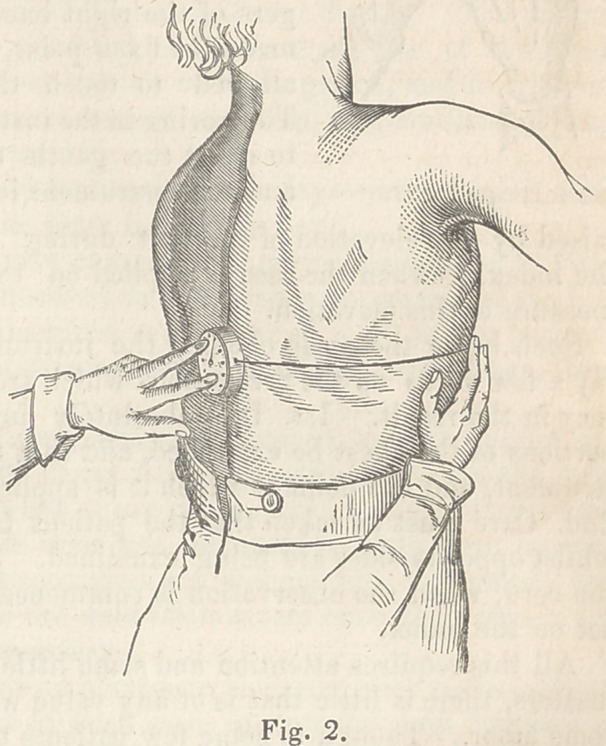


**Fig. 3. f3:**
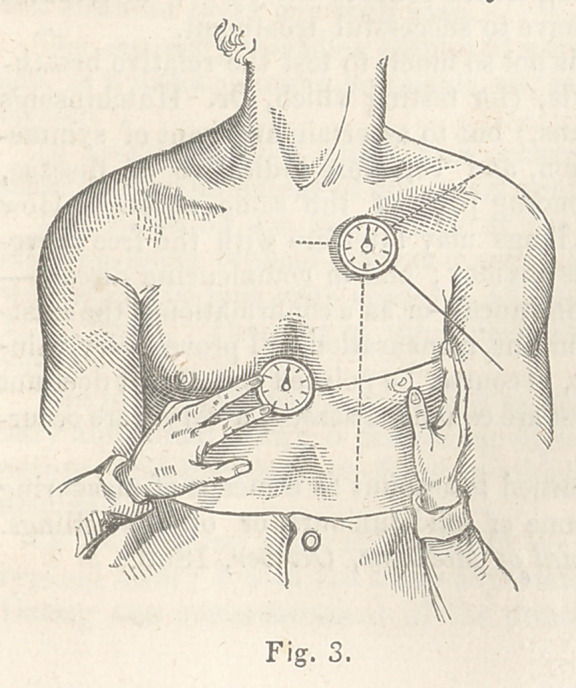


**Fig. 4. f4:**